# Lung Toxicity from Immune Checkpoint Inhibitors: A Diagnostic Approach

**DOI:** 10.3390/jcm14176133

**Published:** 2025-08-29

**Authors:** Ana Casal, Virginia Leiro-Fernández, Laura Villar-Aguilar, Manuel Casal-Guisande, Mar Mosteiro-Añón, Maribel Botana-Rial, Cristina Represas-Represas, María Torres-Durán, Alberto Fernández-Villar

**Affiliations:** 1 Pulmonary Department, Hospital Universitario Álvaro Cunqueiro, 36312 Vigo, Spain; virginia.leiro.fernandez@sergas.es (V.L.-F.); laura.villar.aguilar@sergas.es (L.V.-A.); mar.mosteiro.anon@sergas.es (M.M.-A.); maria.isabel.botana.rial@sergas.es (M.B.-R.); cristina.represas.represas@sergas.es (C.R.-R.); maria.torres.duran@sergas.es (M.T.-D.); jose.alberto.fernandez.villar@sergas.es (A.F.-V.); 2 NeumoVigo I+i Research Group, Galicia Sur Health Research Institute (IIS Galicia Sur), SERGAS-UVIGO, 36312 Vigo, Spain; manuel.casal.guisande@uvigo.gal; 3 Centro de Investigación Biomédica en Red, CIBERES ISCIII, 28029 Madrid, Spain; 4 Fundación Pública Galega de Investigación Biomédica Galicia Sur, Hospital Álvaro Cunqueiro, 36312 Vigo, Spain; 5 School of Industrial Engineering, University of Vigo, 36310 Vigo, Spain

**Keywords:** immune checkpoint inhibitors, lung damage, pneumonitis

## Abstract

The use of immune checkpoint inhibitors (ICIs) has increased exponentially in recent years, leading to a significant impact on cancer patient survival. However, their administration can trigger immune-mediated adverse effects, notably pulmonary toxicity, which is a potentially serious complication. ICI-induced pneumonitis has a variable incidence ranging from 5 to 19% and usually appears in the first few months of treatment. The diagnosis requires a high index of suspicion, especially in patients with risk factors (elderly male smokers with squamous cell lung cancer, previous respiratory or autoimmune disease, and receiving combination treatment with other ICIs or chemo-radiotherapy). Chest computed tomography (CT) is a key test, allowing the identification of different radiological patterns. This study can be completed with bronchoscopy with bronchoalveolar lavage (BAL) to rule out infection or tumour progression. In general terms, treatment is based on discontinuing the causative drug, with or without the initiation of systemic corticosteroids, escalating to immunosuppressants depending on the severity and/or refractoriness of the condition. This paper provides an updated narrative review of ICI pulmonary toxicity, addressing its pathophysiology, different types of lung damage, diagnostic and therapeutic algorithms, and the emerging role of biomarkers such as KL-6 or IL-6. This article emphasises the need for a multidisciplinary approach and further prospective studies to optimise the management and prognosis of this immune-mediated complication.

## 1. Introduction

Immune checkpoint inhibitors (ICIs) are a form of immunotherapy that boosts the patient’s immune response to detect and eliminate cancer cells. The immune system and cancer have a dynamic relationship. The immune system normally recognises and eliminates cancerous cells, but cancer cells can evade this surveillance through genetic changes and by producing immunosuppressive signals. This interplay is so critical that boosting the immune system through cancer immunotherapy has become a revolutionary cancer treatment. Thus, immunotherapy is a medical treatment that harnesses the power of the immune system to fight diseases like cancer by stimulating the immune system to attack specific cancer cells or by suppressing an overactive immune response in conditions like autoimmune diseases.

Since the Food and Drug Administration (FDA) approved ipilimumab for metastatic melanoma in 2011, the use of ICI has grown exponentially, becoming a fundamental treatment for locally advanced and advanced non-small cell lung cancer (NSCLC) without target mutations, and even for small cell lung cancer (SCLC) [1,2,3]. However, despite the considerable improvement in outcomes following the incorporation of ICIs [4], there has been a corresponding increase in complications related to their administration, known as immune-related adverse events (IRAEs), which vary in severity and can affect virtually any organ [5].

Although pneumonitis is a general term used to refer to lung damage of various aetiologies, ICI pneumonitis specifically refers to a non-infectious inflammatory process in the lungs resulting from the administration of an ICI. ICIs can cause pulmonary toxicity because the activation of T cells and proinflammatory cytokines may trigger an exaggerated immune response against lung tissue. The lungs are particularly vulnerable compared to other organs due to their continuous exposure to inhaled antigens, high vascularisation, and delicate alveolar structures, which amplify immune-mediated injury. While immune-related toxicities can occur in many organs, pulmonary toxicity is distinct in that even mild inflammation can critically impair gas exchange and rapidly become life-threatening, making it a unique and clinically significant manifestation.

The incidence of ICI pneumonitis ranges from 5% to 19% [6], being higher with programmed cell death protein 1 (PD-1) inhibitors than with programmed cell death ligand 1 (PD-L1) and cytotoxic T lymphocyte-associated protein 4 (CTLA-4) inhibitors [7]. Despite its clinical relevance, ICI pneumonitis continues to be a diagnostic and therapeutic challenge in daily practise, especially due to its non-specific clinical presentation and because it is a diagnosis of exclusion. The available evidence on this complication is heterogeneous and, in many cases, limited to retrospective studies, clinical cases, and non-systematic reviews. In this context, it is essential to have tools that provide an up-to-date summary of the knowledge on its clinical management.

This paper presents a narrative review with an integrated approach, focusing specifically on ICI-induced pulmonary toxicity. We believe it adds value to previous publications by compiling and structuring the most relevant clinical, radiological, pathophysiological, and therapeutic aspects, including diagnostic algorithms and management proposals, based on guidelines and recent evidence. It also highlights the need for a multidisciplinary approach and points to future lines of research, such as the identification of predictive biomarkers and the personalisation of treatment. In short, this article aims to be a useful tool for both clinicians and researchers in the context of the growing use of immunotherapy in cancer treatment.

### 1.1. Types of Immunotherapy

Immunotherapy is a generic term that encompasses various types of treatments, including ICIs [8]. The most common types of immunotherapy are the following:

- Chimeric antigen receptor T cell therapy (CAR-T): Obtaining T cells from the patient for modification and aggregation of artificial receptors that recognise specific tumour antigens, with the consequent activation of these T cells and selective tumour destruction.
- Monoclonal antibodies (mAbs): There are different types of mAbs with the ability to act on various antigens with the ultimate goal of destroying tumour cells.
- Immune checkpoint inhibitors (ICIs): Responsible for inhibiting immune checkpoints to promote the immune system’s attack on the tumour. They are the most common type of immunotherapy. The most commonly used in lung cancer are the following:
  ○ T cell-associated protein 4 inhibitors (CTLA-4);
  ○ Programmed cell death protein 1 (PD-1) inhibitors;
  ○ Programmed cell death ligand 1 (PD-L1) inhibitors.
  As an example of how ICIs work, checkpoint proteins such as PD-L1 (in tumour cells) and PD-1 (in T cells) play an important role in controlling immune responses. The binding of PD-L1 to PD-1 prevents T cells from becoming “activated” and attacking tumour cells. Therefore, with the administration of ICIs (anti-PD-L1 or anti-PD-1), this PD-L1-PD-1 binding is prevented, keeping T cells active so that they attack tumour cells [8,9].
- Cytokines (CKs): Small proteins that activate the immune system when secreted. The most commonly used are interleukins and interferons.
- Cancer vaccines: Vaccines responsible for activating the immune system so that it can destroy cancer cells.

### 1.2. Signalling Pathways of Lung Damage Caused by Immune Checkpoint Inhibitors

To date, the pathogenesis and specific signalling pathways of lung damage caused by ICIs have been little explored. This complexity is compounded by the fact that in most cases, ICIs are not administered in isolation but in combination with other cancer therapies with potential synergistic and/or toxic effects, making it difficult to identify specific causal mechanisms [10]. As mentioned above, ICIs activate T cells to destroy cancer cells, but this is not a harmless mechanism, as they also alter immune homeostasis, which can lead to adverse effects such as autoimmunity and non-specific inflammation [11].

**Figure 1:**
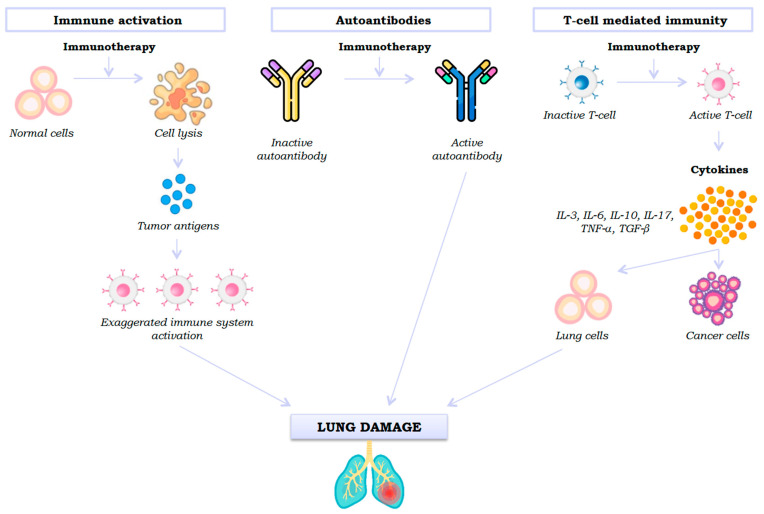
Signalling pathways of lung damage caused by immune checkpoint inhibitors.

Although evidence is scarce, three mechanisms of lung damage signalling by an ICI have been proposed: generalised immune activation by checkpoint neutralisation, pre-existing autoantibodies, and unwanted effects of T cell-mediated immunity (Figure 1).

First, ICIs promote a change in the phenotype of inactive T cells to their active effector state, stimulating the immune system, which leads to the release of cytokines (IL-3, IL-6, IL-10, IL-17, TNF-α, and TGF-β) [12]. However, active T cells not only recognise tumour cells but also shared antigens in healthy lung parenchyma, producing cytotoxic activity. Thus, stimulation of the immune system (Th1 and Th17 lymphocytes) and the production of proinflammatory cytokines will cause inflammation and lung damage [13].

Secondly, pre-existing autoantibodies also appear to play a role in immunotherapy-induced lung damage, although the mechanism is unknown. Recent studies indicate that patients with autoantibodies who do not have an autoimmune disease prior to administration of an ICI develop immune-mediated adverse effects after taking it [14].

Thirdly, another mechanism involved in immunotherapy-induced lung toxicity is the release of tumour antigens produced after cell lysis, which leads to an exaggerated activation of the immune system and consequent pneumonitis [15].

Understanding these pathways is essential for advancing the development of targeted therapeutic strategies, such as the rational use of immunosuppressants, and the identification of biomarkers that would allow for more homogeneous stratification of the risk of pulmonary toxicity.

## 2. Lung Toxicity from Immune Checkpoint Inhibitors

### 2.1. Frequency of Toxicities

Retrospective epidemiological studies report an incidence of lung toxicity from ICIs ranging from 3.5% to 19% depending on the series [6,16,17]. Furthermore, the incidence of pneumonitis varies significantly depending on the type of tumour (higher in NSCLC compared to renal cancer or melanoma), patient risk factors (higher in male smokers with previous lung or autoimmune disease), and the specific type of drug administered (higher for anti-PD-1) (Table 1).

Thus, the classic series describe a higher incidence of pneumonitis after an ICI if the treatment administered is anti-PD1 compared to anti-PD-L1 (3.6% vs. 1.3%, respectively). A recent meta-analysis including 18,715 patients treated with anti-PD-1 or anti-PD-L1 observed an incidence of pneumonitis of 2.8% (95% CI, 2.4–3.2%) [18]. In contrast, the lowest incidence of pulmonary toxicity after an ICI is observed after administration of anti-CTLA-4. Khoja et al. describe an increased risk of pneumonitis if the drug administered is anti-PD-1 compared to anti-CTLA-4 (OR 6.4; 95% CI: 3.2–12.7) [19]. In addition, a higher probability of pneumonitis has been reported in patients receiving combination immunotherapy compared to monotherapy (incidence of 10% vs. 3%, *p* < 0.001) [6]. Despite the data described, the incidence of pulmonary toxicity from ICIs in routine clinical practise remains unknown, and it has been postulated that it may be significantly higher than that reported in clinical trials [20].

### 2.2. Latency Time

The latency period for the onset of pulmonary toxicity after administration of an ICI varies, with onset most frequently occurring within the first 3 months after initiation. However, it should be noted that there are cases of ICI-induced pneumonitis described up to years after the start of treatment [21]. In their multicentre study of patients with stage IV NSCLC and melanoma, Delaunay et al. found that pneumonitis after administration of ICIs occurred at a mean of 2.3 months, with earlier onset in cases of NSCLC (2.1 months) compared to cases of melanoma (5.2 months) (*p* = 0.02) [22].

### 2.3. Grades of Pulmonary Toxicity

Cases of lung toxicity caused by ICIs can vary in severity, and an international grading system should be used to classify them, which will facilitate future comparisons of cases between studies. Table 2 shows the criteria for the five grades of ICI pneumonitis according to the National Cancer Institute Common Terminology Criteria for Adverse Events [23].

## 3. Types of Lung Damage Caused by Immune Checkpoint Inhibitors

In cancer patients, alterations in immune responses induced by ICIs (such as T cell activation, shifts in cytokine profiles, and loss of peripheral tolerance) are closely correlated with both the severity and type of pulmonary toxicity. Enhanced immune reactivity can lead to different histopathological patterns (ICI-related bronchiolitis, sarcoidosis-like granulomatous reaction, and venous thromboembolic disease, among others), each associated with distinct clinical presentations and radiological findings. The intensity of these immune changes often parallels the grade of toxicity, with more pronounced immune dysregulation resulting in higher-grade, potentially life-threatening pulmonary complications.

### 3.1. Pneumonitis Caused by Immune Checkpoint Inhibitors

ICI pneumonitis is a rare condition (estimated incidence of 4% for anti-PD-1, 2% for anti-PD-L1, and <1% for anti-CTLA-4, reaching up to 10% with combination immunotherapy), but is potentially fatal, resulting from inflammation of the lung parenchyma secondary to the administration of these drugs [24]. There are patient risk factors that predispose to ICI pulmonary toxicity, such as smoking, older age, pre-existing lung diseases (such as airway diseases but especially fibrotic interstitial lung disease), concomitant treatments (radiotherapy and/or chemotherapy), and specific histological type of NSCLC (epidermoid) [22]. In addition, these factors have been associated with a poor prognosis for toxic pneumonitis, adding to the severity of the pulmonary condition (greater hypoxemia and/or greater extent of infiltrates), acute onset, comorbidity, previous autoimmune disease, poorer functional status, and greater diagnostic delay [25]. For this reason, it has been postulated that in the specific subgroup of patients with higher risk factors, a high index of suspicion and active case findings should be maintained when initiating ICI treatment.

**Figure 2:**
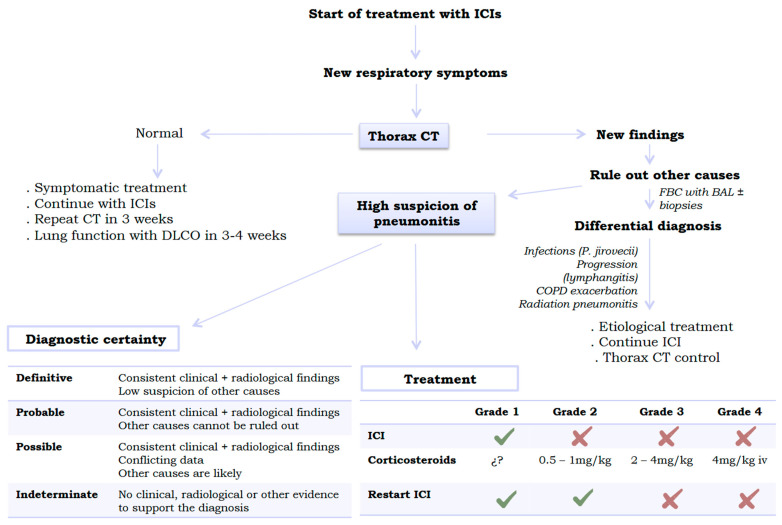
Algorithm for managing suspected pulmonary toxicity caused by immune checkpoint inhibitors. **Legend: CT**, computed tomography; **DLCO**, carbon monoxide diffusion capacity; **FBC**, fibrobronchoscopy; **ICI**, immune checkpoint inhibitor; **BAL**, bronchoalveolar lavage.

The diagnosis of ICI pneumonitis is based on a combination of non-specific respiratory symptoms (dyspnoea, cough, low-grade fever…), a temporal relationship compatible with the onset of treatment, the appearance of new infiltrates on imaging tests (chest CT scan), and the exclusion of other causes (mainly through bronchoscopy with BAL to rule out infection). A diagnostic classification (definite, probable, possible, and indeterminate diagnosis) has been established based on the degree of diagnostic certainty obtained after performing the various complementary tests (Figure 2). Thus, lung biopsy is reserved for cases where other aetiologies cannot be ruled out. Nevertheless, it should be noted that a definitive diagnosis of ICI pneumonitis is rare even when invasive tests are performed (with lung biopsy by needle aspiration or video-assisted thoracoscopy being the most diagnostically useful) [25].

From a radiological point of view, chest CT scans can reveal different types of patterns compatible with ICI pneumonitis, such as organised pneumonia (the most common), ground-glass opacities, hypersensitivity pneumonitis, non-specific interstitial pneumonia, eosinophilic pneumonia, or acute alveolar damage. However, there are also other less common imaging patterns (usual interstitial pneumonia, vasculitis, proteinosis, veno-occlusive disease, or constrictive bronchiolitis) [26]. Similarly, at the histopathological level, several findings compatible with ICI pneumonitis have been described, such as cellular interstitial pneumonitis, chronic interstitial inflammation, granulomatous inflammation, organising pneumonia, and diffuse alveolar damage; there is also a significant frequency of mixed patterns [19].

### 3.2. “Sarcoidosis-like” Granulomatous Reaction

The sarcoidosis-like granulomatous reaction is a rare and underdiagnosed type of pulmonary toxicity caused by immunotherapy, as it is often confused with cancer treatment failure or tumour progression due to misinterpretation of imaging findings [27]. Sarcoidosis-like not only affects the lungs but other commonly affected organs as well, including the skin and lymph nodes. Thus, immunotherapy-induced sarcoid granulomas have been described after administration of ipilimumab, nivolumab, and pembrolizumab, with a variable incidence that is probably underestimated for the reasons explained above [28].

A diagnosis of sarcoidosis-like is suspected when a chest CT scan is compatible (micronodular pulmonary disease, ground-glass infiltrates with subpleural or perihilar distribution, and/or enlarged hilar–mediastinal lymph nodes), and a suggestive bronchoalveolar lavage (lymphocytosis with increased CD4/CD8 ratio), and is confirmed by compatible histology (non-necrotising granulomas) after ruling out other causes. Thus, at the tissue level, sarcoidosis-like disease is characterised by an immune reaction that induces granulomas with epithelioid cells surrounded by a ring of CD4+ and CD8+ T lymphocytes [29].

The indications for treatment of sarcoidosis-like disease are not clearly defined, but treatment with corticosteroids is recommended in cases of radiological stage ≥II sarcoidosis, incapacitating symptoms, involvement of vital organs, and radiological and/or functional progression. Overall, the prognosis is good, with regression of symptoms after discontinuation of the ICI, with or without the use of corticosteroids. In addition, sarcoidosis-like disease has been associated with longer response rates to cancer treatment [22].

### 3.3. Venous Thromboembolic Disease

Knowledge of cardiovascular complications associated with ICIs is limited, and there are few studies that specifically analyse venous thromboembolic disease (VTD) caused by ICIs, given that this risk may be increased not only by immunotherapy but also by predisposing factors such as cancer itself, immune activation, or combination with other cancer treatments [30,31]. Therefore, the actual incidence of VTD following administration of an ICI is unknown. A study of 672 patients treated with an ICI observed 47 episodes of VTE with a cumulative incidence of 12.9%, and another single-centre study of 228 patients with melanoma treated with an ICI identified episodes of VTD in 16.2% of the study subjects [32,33]. The management of VTD caused by ICIs does not differ from VTD caused by other factors. In this case, anticoagulation is recommended while the cancer is active, and indefinite anticoagulation should be considered subsequently, depending on the specific characteristics of each patient. In cases of grade 1 to 3 VTD, continuing with ICIs may be considered, while in cases of grade 4 VTD, definitive discontinuation of treatment is recommended [34].

### 3.4. Other Types of Lung Damage Caused by Less Common Immune Checkpoint Inhibitors

#### 3.4.1. Bronchiolitis Caused by ICIs

Respiratory bronchiolitis following ICIs is a less common and less well-defined type of pulmonary toxicity, as it has only been reported in case series [35]. Classically, bronchiolitis is characterised on chest CT by centrilobular nodules, thickening of the bronchial walls, consolidations, ground-glass opacities, and/or a tree-in-bud pattern. For a correct diagnosis, it must be ruled out that these radiological alterations are due to infectious, aspirational, or other inflammatory causes. Thus, suspicion of this entity should be raised in the absence of infectious symptoms and confirmed by imaging tests documenting the resolution of symptoms after maintenance treatment or trial with corticosteroids [36].

#### 3.4.2. Diaphragmatic Myositis Due to ICIs

There is little evidence regarding other types of pulmonary toxicity caused by ICIs, such as diaphragmatic myositis. There have been reports of case series of myositis following administration of ICIs, both as a monotherapy and in combination. Haddox et al. describe a case of severe necrotising diaphragmatic myositis that resulted in fatal progressive hypoventilation following treatment with anti-PD-1 [37]. John et al. published another case of fatal progressive hypoventilation due to diaphragmatic myositis in a patient who had received combination therapy (anti-CTLA-4 and anti-PD-1) [38].

#### 3.4.3. Tuberculosis Caused by ICIs

There is little evidence on the incidence of tuberculosis after starting treatment with an ICI. Some case series have described the reactivation of tuberculosis in patients treated with anti-PD-1 and suggested an immune reactivation syndrome [39].

#### 3.4.4. Allergic Bronchopulmonary Aspergillosis Caused by ICIs

Allergic bronchopulmonary aspergillosis following ICI administration is poorly understood. Pradere et al. published a case of diagnosed allergic bronchopulmonary aspergillosis treated with immunotherapy due to elevated serum immunoglobulin E (up to 2514 kU/L) and the presence of positive IgE antibodies against Aspergillus fumigatus. This case was treated with antifungals (itraconazole) and corticosteroids, with resolution of the condition [40].

## 4. Diagnostic Approach to Lung Toxicity Caused by Immune Checkpoint Inhibitors

For the correct diagnosis of ICI pulmonary toxicity, it is necessary to maintain a high index of suspicion given that the respiratory symptoms are non-specific. Therefore, the correct diagnosis of lung toxicity due to ICIs will follow a series of guiding steps: (1) newly appearing pulmonary infiltrates in imaging tests, normally bilateral and non-segmental in distribution; (2) a compatible temporal relationship between the start of treatment with the ICI and the onset of symptoms; and (3) exclusion of alternative causes.

As mentioned above, given that pneumonitis is more common in the first few months of ICI treatment, extra vigilance is required during this period. If pulmonary toxicity due to the ICI is suspected, imaging tests (initial chest X-ray and, subsequently, chest CT scan) are indicated in order to make a correct differential diagnosis (tumour progression, infection, pulmonary thromboembolism…) and thus guide the rest of the complementary diagnostic tests. Thus, after performing a chest CT scan, bronchoscopy with bronchoalveolar lavage (which, in the case of ICI pneumonitis, would be predominantly lymphocytic) would be indicated if the patient’s clinical condition allows it, primarily to rule out infectious aetiology. Lung biopsies may be indicated in cases where other causes (such as tumour progression) cannot be ruled out despite the aforementioned complementary tests [22].

The recommendations of the European Society for Medical Oncology (ESMO) establish surgical lung biopsy by video-assisted thoracoscopy as the gold standard in patients who require a histological lung sample to ensure diagnostic certainty [34]. However, the clinical practise guidelines of the American Society of Clinical Oncology (ASCO) are more conservative and recommend endoscopic transbronchial biopsies in cases requiring an anatomopathological sample [41]. Therefore, given that there is no clear consensus, the indication for invasive procedures should be discussed in a multidisciplinary meeting (including pulmonologists, oncologists, thoracic surgeons, pathologists, and radiologists). Figure 2 shows the diagnostic approach algorithm for suspected lung toxicity due to ICIs.

## 5. Management of Lung Damage Caused by Immune Checkpoint Inhibitors

**Figure 3:**
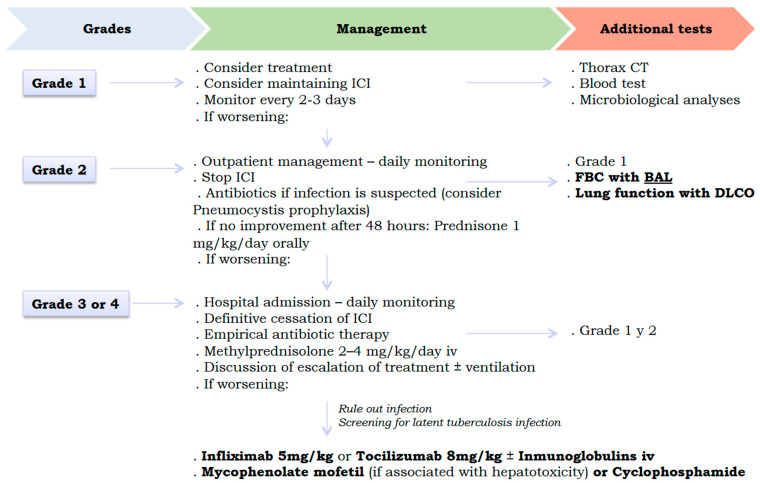
Therapeutic algorithm for lung toxicity caused by immune checkpoint inhibitors. **Legend: CT**, computed tomography; **DLCO,** carbon monoxide diffusion capacity; **FBC**, fibrobronchoscopy; **ICI,** immune checkpoint inhibitor; **BAL,** bronchoalveolar lavage.

Respiratory toxicity caused by ICIs can have devastating and potentially irreversible consequences (such as pulmonary fibrosis) if not managed early [42]. Therefore, early action to discontinue the causative drug will improve the prognosis for patients. Figure 3 shows the proposed therapeutic algorithm for pulmonary toxicity caused by ICIs. International consensus (National Comprehensive Cancer Network, American Society of Clinical Oncology, Society for Immunotherapy of Cancer, and European Society for Medical Oncology) proposes discontinuing ICIs at grade 2 pulmonary toxicity, with no possibility of reintroducing it (after resolution of the condition) in cases of grade 3 or higher. In addition, it is recommended to start corticosteroid therapy at grade 2 (higher doses of corticosteroids for higher grades), with the possibility of escalating to biological and/or immunosuppressive therapy at grade 3 if there is no response to corticosteroids [34,41,43,44,45]. Thus, the drugs available in the latter case would be infliximab (anti-TNF-α), tocilizumab (anti-IL6), intravenous immunoglobulins, mycophenolate mofetil (purine synthesis inhibitor in lymphocytes, especially in cases with associated hepatotoxicity), and cyclophosphamide [41,43,44,45]. The specific use of these drugs is deduced in order to neutralise the cytokines involved in the ICI lung damage signalling pathways previously explained (IL-6, IL-10, IL-17, TNF-α and TGF-β, …). These treatments should also be combined with the necessary supportive measures for each case, such as supplemental oxygen, non-invasive and invasive mechanical ventilation, and prophylactic antibiotic therapy. It should be noted that these therapeutic recommendations are based on retrospective studies and expert opinions, as no clinical trials have been conducted to demonstrate the efficacy of glucocorticoids and other alternative therapies in cases of lung toxicity due to ICIs [46].

## 6. Resolution Time

A favourable prognosis is reported following ICI-induced pulmonary toxicity, especially if early and appropriate management is provided. Overall, the estimated time for resolution of ICI-induced pulmonary damage is between 6 and 10 weeks [22]. It should be noted that in cases where reintroduction of an ICI is indicated after a case of pneumonitis (grades 1 and 2), there is a possibility of recurrence, estimated at between 17% and 29%, depending on the series, and therefore, it is not indicated to change the specific type of ICI for a different one [34,45].

## 7. Effectiveness Markers

Retrospective studies indicate higher rates of cancer control in patients who experienced immune-related adverse effects [47]. Pawel et al. observed that patients who received anti-PD-L1 treatment and experienced immune-related adverse effects had higher overall survival compared to cases without toxicity (CRI 0.79; 95% CI: 0.60–1.05) [48]. Sato et al. observed that patients with advanced NSCLC treated with anti-PD-1 who had immune-mediated adverse effects had a higher objective response rate (63.6% vs. 7.4%, *p* < 0.01) and longer progression-free survival (HR 0.10; 95% CI 0.04–1.46; *p* = 0.13) compared to those who did not have immune-related adverse effects [49]. The exact mechanisms explaining how immune-related toxicity can be a subsequent marker of efficacy are unknown. It has been postulated that patients who respond to immunotherapy are those who actually have longer survival and, therefore, a longer treatment duration and consequently more time to develop immune-related adverse events [50].

On the other hand, it is worth mentioning the importance of researching future biomarkers as potential indicators of efficacy. In this regard, there have been promising advances in the use of serum biomarkers such as KL-6 (Krebs von den Lungen-6), which is a glycoprotein expressed by type II pneumocytes in response to cell damage. There is evidence linking increased circulating levels of KL-6 with greater interstitial lung toxicity [51]. Another potential biomarker in lung cancer patients treated with ICPI is IL-6. Naqash et al. report that C-reactive protein and IL-6 levels are elevated in ICI pneumonitis following treatment with atezolizumab [52]. Similarly, recent studies have observed that the presence of certain pre-existing antibodies is associated with the development of immune-mediated adverse effects in NSCLC patients treated with nivolumab or pembrolizumab, although the mechanism of action remains unclear [53]. Therefore, further studies are needed to define the role of these biomarkers in the diagnosis and monitoring of ICI-induced pulmonary toxicity.

## 8. Conclusions

Pulmonary toxicity associated with treatment with immune checkpoint inhibitors is a significant immune-mediated complication with a non-negligible incidence, especially during the first months of treatment. Although respiratory symptoms are usually non-specific, radiological findings can guide diagnosis, particularly in patients with known risk factors. This paper provides an overview of the main types of ICI-related pulmonary toxicity, addressing pathophysiological, diagnostic, and therapeutic aspects based on current scientific evidence. In this context, it is essential to maintain a high index of suspicion with at-risk patients (older male smokers with squamous cell lung cancer, respiratory or autoimmune comorbidity, and exposure to anti-PD1, combinations of immunotherapy, or previous thoracic radiotherapy) to facilitate early diagnosis and treatment that could improve prognosis. Fibrobronchoscopy with bronchoalveolar lavage plays a key role in ruling out infections or other alternative causes, and lung biopsy, when indicated, can provide relevant pathological information, such as the presence of lymphocytic alveolitis. Despite advances, gaps in knowledge remain, especially regarding the chronology of the onset of adverse effects, their pathophysiology, and the predictive value of biomarkers, so further studies are needed. In short, this article aims to contribute to structuring the clinical approach to these complications, support decision-making, and stimulate future lines of research in this growing field of treatment.

## Figures and Tables

**Table 1:** Risk factors for lung damage caused by immune checkpoint inhibitors.

**Demographic factors**	. Male sex . Older age . Worse functional status . Greater degree of dependence for activities
**Previous diseases**	. Respiratory disease: Asthma, ILD, ILA, COPD, PH . Autoimmune disease
**Toxic habits**	. Smoking . Other inhaled toxics
**Type of tumour**	. NSCLC (epidermoid)
**Treatment regimen**	. Combination of ICI . Previous radiotherapy
**Type of ICI**	. Higher-risk anti-PD1 . Low-risk anti-CTLA-4

**Legend**: COPD: chronic obstructive pulmonary disease; **ICI:** immune checkpoint inhibitor; **ILA:** interstitial lung abnormality; **ILD:** interstitial lung disease; **NSCLC:** non-small cell lung cancer; **PH:** pulmonary hypertension.

**Table 2:** Grades of pneumonitis (National Cancer Institute Common Terminology Criteria for Adverse Events).

Grade	Definition
**1**	. Asymptomatic . Radiological abnormalities: ground-glass opacity, NSIP…
**2**	. Mild/moderate symptoms: shortness of breath, cough, chest pain . Radiological abnormalities
**3**	. Severe symptoms/signs: dyspnoea, respiratory failure . Radiological abnormalities
**4**	. Respiratory symptoms with vital compromise: ARDS . Radiological abnormalities
**5**	. Death related to the adverse effect

**Legend: NSIP:** non-specific interstitial pneumonia; **ARDS**: acute respiratory distress syndrome.
